# Hirayama Disease in an Adolescent Male With Right Hand Weakness and Muscle Wasting

**DOI:** 10.31486/toj.19.0045

**Published:** 2020

**Authors:** Julian Llano, Neil U. Lall, Lamar Davis, Andrew J. Steven

**Affiliations:** ^1^ Universidad CES, Medellín, Colombia; ^2^ Department of Radiology, Emory University School of Medicine, Atlanta, GA; ^3^ Department of Pediatrics, Ochsner Clinic Foundation, New Orleans, LA; ^4^ Department of Radiology, Ochsner Clinic Foundation, New Orleans, LA; ^5^ The University of Queensland Faculty of Medicine, Ochsner Clinical School, New Orleans, LA

## INTRODUCTION

Hirayama disease, also known as monomelic amyotrophy^1^ or juvenile muscular atrophy of the upper extremity,^2,3^ is a rare neurologic condition that involves the inferior motor neurons^1^ and commonly affects the C7 to T1 spinal nerves and their myotomes.^4^ Hirayama first described this type of cervical myelopathy in 1959, stating that it was clinically differentiated from the other known types of motor neuron disease.^5^

Characterized by insidious onset,^1,6,7^ this disease, commonly associated with Asian descent, has a male predominance ranging from 7:1^4,6^ to 20:1,^7,8^ depending on the population, and is typically diagnosed during the second and third decades of life.^1,6,8,9^ Although the exact pathophysiology is unknown, one theory is an imbalance between the growth of the vertebral column and that of the spinal canal contents,^7^ a discrepancy that results in forward displacement of the posterior dural sac with neck flexion,^10^ leading to compression and injury of the spinal cord with posterior damage to the anterior horn cells.^6^ Hirayama disease commonly presents with unilateral or bilateral asymmetric weakness and muscle wasting of the C7 to T1 myotomes, with characteristic sparing of the brachioradialis muscle and without associated sensory loss.^6^

## HISTORY AND CASE REPORT

A 14-year-old male with no significant medical history presented to the pediatric neurology clinic complaining of 7 to 8 months of progressive inability to straighten his wrists. He noted problems with his handwriting, holding utensils, texting, and manipulating the joysticks of his Xbox controller. The patient additionally reported having cramps, numbness, tingling, and shooting pain in his hands associated with neck flexion. The patient's mother described an episode of neck trauma that had occurred approximately 1 year prior to presentation, but the patient had had no significant symptoms at that time.

Physical examination revealed atrophy of the hypothenar eminence and the first dorsal interosseous muscle bilaterally. Weakness was present in both upper extremities, asymmetrically affecting the right side. Additionally, a very low amplitude tremor was elicited with finger extension, consistent with minipolymyoclonus. No sensory deficits were identified, and no abnormal findings were noted in the examination of the lower extremities.

## RADIOGRAPHIC APPEARANCE AND TREATMENT

Nerve conduction studies (NCSs) revealed reduction of the compound muscle action potential (CMAP) amplitudes of the bilateral ulnar nerves. Electromyography (EMG) showed active denervation of the right dorsal interosseous and questionable denervation in the right triceps, suggesting a chronic neurogenic process affecting primarily C8 and T1 on the right with partial C7 involvement.

**Figure 1.**
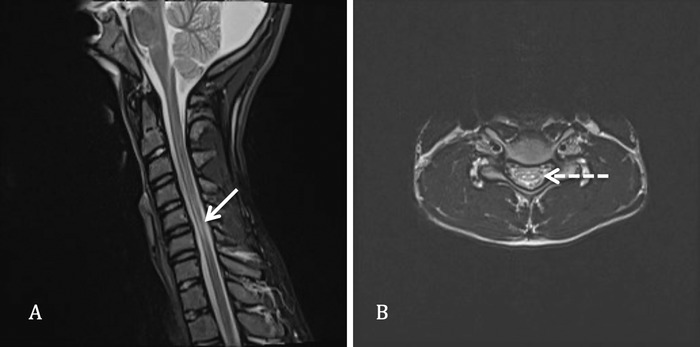
**Routine neutral position cervical spine magnetic resonance imaging. (A) Sagittal T2-weighted image shows thinning of the spinal cord at the C5-C6 level (white arrow). (B) Axial T2-weighted image shows 2 foci of central paramedian hyperintensity, also known as snake-eye appearance (dashed arrow).**

Initial cervical spine magnetic resonance imaging (MRI) showed thinning of the cervical cord centered at the C5-C6 level. Small foci of increased T2 signal intensity were present within the central aspect of the cord in the region of the anterior horn cells extending from C3 to C7 (Figure 1). No associated compressive lesion was identified in neutral position.

**Figure 2.**
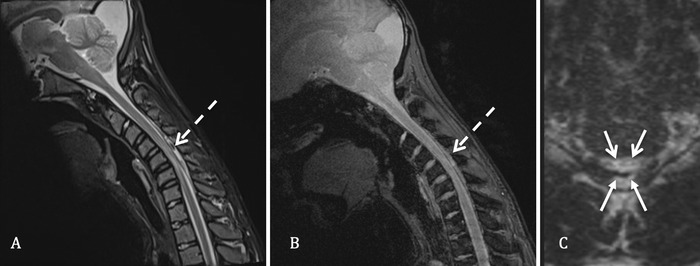
**Flexion position cervical spine magnetic resonance imaging**. (A) Sagittal T2-weighted short tau inversion recovery (STIR) image and (B) sagittal T2*-gradient recalled echo (GRE) image reveal expansion of the posterior cervical epidural space with hyperintense fluid signal and T2 hypointense epidural flow voids from C3 to C7 (dashed arrows in views A and B), ventral displacement of the dura mater, and compression of the spinal cord. (C) Axial 3-dimensional T2 image shows a compressed thecal sac with the classic snake-eye appearance due to compression of the spinal cord (upper arrows). The posterior cervical epidural space is also enlarged with fluid signal and anterior displacement of the dura mater (lower arrows).

The patient returned for additional MRI with flexion positioning. Flexion views showed expansion of the posterior epidural space from C3 through C7, measuring up to 0.5 cm in thickness. The ventral dural displacement resulted in complete effacement of the cerebrospinal fluid within the spinal canal and localized mass effect on the spinal cord (Figure 2). The appearance confirmed the diagnosis of Hirayama disease.

The patient was treated conservatively with a cervical collar to be worn at all times, and a follow-up MRI was scheduled 3 months later.

## DISCUSSION

Hirayama disease, although a rare entity, should be part of the differential diagnosis for young male patients^11^ complaining of upper extremity weakness associated with muscle wasting. Although familial presentations have been described, the disease is typically sporadic as in this case.^3,8,9^

Because Hirayama disease is a rare disease, its etiology and pathophysiology are not well understood. The cervical myelopathy appears to result from anterior displacement of the posterior cervical dura compressing the spinal cord against the vertebral bodies.^6,10^ This displacement is theorized to be caused by an imbalance between the growth of the vertebral column and the growth of the spinal canal contents.^7^ Another theory is that the increased laxity of the dura mater, anchored superiorly at C2-C3, allows for increased cord movement and concomitant anterior displacement with neck flexion, resulting in repetitive cervical microtrauma that leads to microischemia of the anterior horn cells.^12^

Hirayama disease often has an insidious onset of progressive weakness and muscular wasting of the upper extremity.^6,9,13^ The disease appears to be self-limiting, reaching a stable state approximately 3 to 5 years after its onset,^4,14^ a characteristic that differentiates this entity from other known motor neuron diseases.^8^ Although it is often unilateral, affecting the left side more than the right,^15^ Hirayama disease also presents bilaterally (symmetrically or asymmetrically) in 10% of cases.^6,12,16^ Our patient's bilateral, right-sided-predominant symptomatology was not the usual presentation. Typically, the weakness is in the C7-T1 myotome distribution,^6^ affecting extension, flexion, abduction, and adduction of the fingers and wrist, as well as pronation and supination of the forearm.^17^ Our patient's weakness was with flexion and extension of the fingers, flexion of the wrist, and extension of the elbow. The atrophy accompanying the muscle weakness, sometimes referred to as oblique amyotrophy,^7,18^ has a characteristic distribution. It often affects the hand and the ulnar forearm, including the thenar, hypothenar, and interosseous muscles,^17^ and distinctively spares the brachioradialis muscle.^6,8,9^ Our patient had atrophy of the hypothenar region and first distal interosseous muscle without the characteristic oblique amyotrophy. Aside from muscle weakness and atrophy, patients can present with tremor-like movements, usually irregular and coarse, of one or a few fingers.^17^ Symptomatology can worsen with flexion of the neck^15^ and sometimes with cold temperatures, commonly referred to as cold paresis.^12,19^ Our patient presented with a very low amplitude tremor of the fingers and with cramps in his right hand, the latter occurring in approximately 30% of patients with Hirayama disease.^15^

MRI and electrophysiologic studies are usually needed for an accurate diagnosis.^6,12^ NCSs reveal low ulnar CMAPs that are reduced to a greater extent than those recorded from the median nerve,^6,16^ as in our patient, along with borderline CMAP amplitude in the left median nerve. Conduction velocity and sensory studies are normal. EMG shows denervation most commonly in C8 to T1 innervated muscles; C7 innervated muscles may show partial involvement. C5-C6 muscles are typically spared and have a normal needle examination. These findings were seen in our patient.

Routine neutral supine cervical spine MRI may appear normal or may reveal nonspecific myelomalacia in the lower cervical spine. As with the symptomatology, the myelomalacia may be asymmetric, typically involving the left more than the right,^15,20^ with the appearance on axial T2 images commonly referred to as a snake-eye appearance.^13^ Cervical spine MRI with flexion^21^ positioning is critical for confidently establishing the diagnosis. With flexion, anterior shifting of the posterior dura mater becomes evident^10,22^ with enlargement of the posterior cervical epidural space, as well as associated mass effect on the spinal cord compressed anteriorly against the vertebral bodies and typically most pronounced at the level of C5-C6.^6,20,23^ Our patient had symmetric myelomalacia centered at C5-C6 with a central snake-eye appearance on neutral positioning MRI, and flexion MRI showed expansion of the posterior cervical epidural space with ventral dural displacement and mass effect on the spinal cord.

Hirayama disease has a relatively good prognosis compared to other motor neuron diseases,^15^ but currently no treatment is available to reverse the weakness.^6^ Conservative therapy in the form of a cervical collar is typically employed to control progressive symptomatology by preventing the repetitive injury to the spinal cord^12^ with neck flexion. Patients may use the collar for 3 to 4 years.^12^ Other mainstays for management are physical and occupational therapy. Surgical fusion has been used in cases refractory to conservative treatment,^7,12,23^ with the same objective of preventing repetitive neck flexion.^23^ Cervical duraplasty is an alternative treatment for the disease, as it prevents the abnormal forward displacement of the posterior dura mater while preserving flexion of the cervical spine.^24^ As is the norm, our patient was prescribed a surgical collar for conservative treatment, with neurosurgical follow-up scheduled to assess his response.

## CONCLUSION

Hirayama disease is characterized by compression of the cervical spinal cord by the posterior dura during flexion, resulting in damage to the anterior horn cells. Hirayama disease should be considered in the differential diagnosis for patients in their second or third decade of life with weakness and muscle wasting of the upper extremities, especially males. The application of nonconventional MRI techniques, specifically flexion views, is a key component of establishing the diagnosis and differentiating this disease from other motor neuron pathologies. Immobilization is required to halt disease progression.

## Notes

This article meets the Accreditation Council for Graduate Medical Education and the American Board of Medical Specialties Maintenance of Certification competencies for Patient Care and Medical Knowledge.
